# Buruli ulcer disease: challenges and opportunities for Institut Pasteur International Network

**DOI:** 10.1186/1753-6561-5-S1-L5

**Published:** 2011-01-10

**Authors:** Sara Eyangoh

**Affiliations:** 1 Mycobacteriology Service, Centre Pasteur of Cameroon, Yaounde, Cameroon

## 

Buruli ulcer is a neglected tropical disease caused by *Mycobacterium ulcerans*. It is the third most prevalent mycobacterial disease in the world. The disease has been reported in over 30 ountries, mainly in tropical and subtropical regions of Africa and has often been associated with swampy areas. This flesh-eating disease imposes a harsh reality on its victims who endure prolonged periods of suffering. Major challenges to the prevention and control of Buruli ulcer disease are the lack of knowledge surrounding the reservoir and route of transmission of *M. ulcerans*, lack of diagnostic tests that can easily be performed in rural areas, and limited treatment options.

During this decade, the Institut Pasteur International Network developed interest on Buruli ulcer, bringing together scientists from around the network and other research institutes, and allocate funds. It is in this way that a multidisciplinary effort was undertaken in Centre Pasteur of Cameroon on epidemiological and environmental factors associated with *M.ulcerans* to understand the mode of transmission.

Our first research work focused on risk factors and outlined protective factors such as the use of bed nets and the proper care of skin lesions with antiseptic solutions. These results were then used to reinforce education in a basic public health measures by awareness raising campaign. Our more recent studies assessing the role of water bugs as *M. ulcerans* host and/or vector in environmental context provided a first full picture in terms of biodiversity and seasonal and regional dynamics link with variation in the insect tissue colonization rate.

Furthermore, the Centre Pasteur of Cameroon initiated an international course on Microbiology of *M. ulcerans*. This course brought together scientists from 11 African endemic countries and provided knowledge for PCR detection of *M. ulcerans,* the most sensitive and quickest tool available for diagnosis and in tracing environmental sources of *M.ulcerans*. The Centre Pasteur takes advantage of this competence to serve also at country level, as Reference Laboratory for the National BU control programme; it provides diagnostic support for cases confirmation and expertise in different Public health interventions.

In conclusion, within Institut Pasteur International Network there is interest on Buruli ulcer, but more could be done; there are many opportunities to contribute from scientific point of view.

